# Association between androgen receptor gene and behavioral traits in cats (***Felis catus***)

**DOI:** 10.1371/journal.pone.0324055

**Published:** 2025-05-28

**Authors:** Yume Okamoto, Madoka Hattori, Miho Inoue-Murayama

**Affiliations:** 1 Wildlife Research Center, Kyoto University, Kyoto, Japan; 2 Kyoto City University of Arts, Kyoto, Japan; University of Bari, ITALY

## Abstract

Cats (*Felis catus*) are companions familiar to people worldwide. Despite their popularity, few studies have investigated the genetic background of their behavior. This study aimed to explore the relationship between candidate gene genotypes and behavioral traits in cats. Behavioral traits were assessed by cat owners using the Feline Behavioral Assessment and Research Questionnaire (Fe-BARQ), comprising 23 categories. The target gene was the androgen receptor gene (*AR*) associated with behavioral phenotypes such as aggressiveness across species. Specifically, the polymorphism of glutamine repeats within the *AR* exon 1 region was analyzed in 280 neutered/spayed mixed-breed cats (145 males and 135 females), revealing eight alleles with 15–22 repeats. These alleles were categorized into two groups based on the median: short (≤18 repeats) and long (≥19 repeats) types. Generalized linear model analysis revealed that cats carrying the short types displayed higher “purring” scores. Furthermore, male cats with short-type alleles showed higher “directed calls/vocalizations” scores, whereas females with short-type alleles showed higher “stranger-directed aggression” scores, than their respective counterparts with long-type alleles. Additionally, the comparative analysis of the homologous regions of felid *AR* genes revealed that long alleles with 20–22 repeats were specific to cats. This finding suggests that cats with a strong tendency to receive human care from birth may survive without vocal communication, leading to an increase in the frequency of the long alleles. This study provides the first evidence that *AR* glutamine repeats may be associated with specific behavioral traits in cats, and the findings have potential applications in improving animal welfare by predicting behavioral tendencies based on genetic data.

## Introduction

Cats (*Felis catus*) are popular companion animals that play an important role in society through their relationships with humans. Although most Felidae species, including the African wildcat (*Felis lybica*), the cat’s ancestor, are solitary, domestic cats can live in groups and display social behaviors. They use various behaviors and signals, including olfactory, tactile, visual, and vocal, to communicate with humans and other cats [1]. In olfactory communication, cats rely on gland secretions, pheromones, and behaviors such as scratching and use saliva, urine, and feces to convey scent information; actions such as allorubbing, object scratching, and urine spraying also transmit scent signals [2]. Tactile communication involves physical contact, such as allogrooming and rubbing, which express friendliness or comfort as part of social interactions [1]. Visual communication is conveyed through body posture, tail-up position, ear angles, and other body signals [3]. Vocal communication includes a wide range of sounds and is used for affiliative contact, reproduction, solicitation, and antagonistic interactions [4].

Purring, a unique vocalization in cats, plays a key role in vocal communication both in affiliative and solicitation contexts [4]. Initially, purring may serve to signal good health to the mother but continues to aid communication among cats beyond lactation [5]. In affiliative contexts, purring fosters security and comfort, expresses friendliness, and invites play; however, its exact function remains unclear [5]. In addition, purring may serve to avoid conflicts. For example, low-ranking or sick cats may purr when approached by high-ranking individuals [5]. A “solicitation purr” includes a “high-frequency component,” which sounds more urgent and less pleasant to humans than a non-solicitation purr [6]. This variation likely signals a sense of urgency, often to attract attention or requesting food or interaction. Thus, purring apparently is an important behavior for conveying various intentions during vocal communication.

Behavioral traits have been explored extensively as reflections of both “nature” and “nurture,” highlighting the interplay of genetic and environmental factors in shaping animal behavior. Genes can influence behavioral traits more substantially than environmental factors. For instance, genome-wide association studies (GWAS) in dogs (*Canis lupus familiaris*) demonstrated the impact of genetic background on behavior. Genetic variation accounted for over 25% of several behavioral traits in dogs, such as “human sociability,” “toy-directed motor activity,” and “biddability” [7]. Additionally, candidate genes in dogs have been linked to traits such as boldness or sociability [8,9]. Although the genetic-behavioral link in dogs has been investigated extensively, the genetic basis of behavior in cats remains understudied. To date, only two candidate genes, oxytocin receptor (*OXTR*) and arginine vasopressin receptor 1A (*AVPR1a*), have been investigated in relation to feline behavior [10–12]. Understanding the genetic basis of cat behavior can be applied for the welfare of Felidae species. Similarities have been observed in personality structures across Felids, and subjective well-being correlates with personality traits [13,14]. Observing wild animals is often challenging, genetic information can be noninvasively obtained from fecal or hair samples. Therefore, using such genetic data to estimate behavioral traits may offer a practical method for supporting conservation efforts.

The androgen receptor gene (*AR*), located on the X chromosome, encodes a receptor with high affinity for androgens, such as testosterone and dihydrotestosterone. Furthermore, the N-terminal domain of *AR*, which contains the exon 1, is highly conserved among mammals [15]. In humans, the receptor consists of three functional domains encoded by eight exons: the transactivation domain (exon 1), DNA-binding domain (exons 2 and 3), and ligand-binding domain (exons 4–8) [16]. The relationship between *AR* genotypes and phenotypes has been studied previously, with a focus on microsatellite polymorphisms in exon 1. In humans, two microsatellite polymorphisms have been identified, both of which are associated with various traits, such as violent criminal behavior and aggression [17,18]. One polymorphism involves a glutamine repeat region encoded by the CAG sequence in exon 1 that is conserved across several species. Animal studies have linked this polymorphism to behavioral traits such as aggression in dogs and fear responses in camels [19,20]. Although this glutamine repeat polymorphism has also been reported in the cat *AR* gene [21–23], its potential association with behavioral traits is yet to be explored.

This study aimed to investigate the association between behavioral traits and *AR* polymorphisms in cats and explore potential differences between domesticated cats and wild felids.

## Materials and methods

### Ethics statement

DNA sample collection was approved by the research ethics committee and conducted according to the guidelines for the ethics of animal research established by the Wildlife Research Center of Kyoto University (approval numbers WRC-2022-010A, WRC-2022-017A, WRC-2023-010A, and WRC-2023-017A). The questionnaire survey was approved by the research ethics committee and conducted under the guidelines for the ethics of research involving human participants established by the Wildlife Research Center of Kyoto University (approval number WRCH-2022-001). Participants (cat owners who answered the questionnaire) were recruited from September 27, 2022, to June 18, 2023. The purpose of the study was explained to the participants, and written informed consent was obtained. No minors were included in the study.

### Subject animals

Behavioral ratings were collected from 441 cats (241 males and 200 females) with permission from their owners. All cats were kept as pets in their owners’ homes, and 430 cats (236 males and 194 females) were neutered/spayed. Mixed breeds accounted for 86% of all samples (*n* = 380). Genomic DNA was extracted from buccal cell samples obtained from 417 cats (225 males, 192 females). To minimize the effects of breed and neutering/spaying, the analysis was limited to neutered/spayed mixed breeds. In cases where owners had multiple cats rescued from the same city, only one cat was included to avoid closely related animals. After filtration, 280 cat samples (145 males and 135 females) were used for further analysis of *AR* (S1 Dataset).

### Genotyping

Genomic DNA was extracted from buccal cell samples using the DNeasy® Blood & Tissue Kit (QIAGEN, Hilden, Germany). A fragment of *AR* exon 1 containing a CAG repeat encoding glutamine was genotyped. Polymerase chain reaction (PCR) was performed in a 15 μL reaction mixture containing genomic DNA, LA Taq, dNTP mixture, GC Buffer I (TaKaRa, Kusatsu, Shiga, Japan), with the following primers: F, 5′-GCCAGCACCACCGGACGAGAATGA-3′ and R, 5′-TAACTGTCCTTGGAGGAGGTGGAAGCA-3′ [22,24]. The PCR cycle conditions were as follows: initial preheating at 95°C for 2 min, followed by 35 cycles of 95°C for 2 min, 95°C for 30 s, 65°C for 1 min, and 74°C for 2 min, with a final extension at 74°C for 10 min [22]. Following PCR amplification, the amplified products were purified using the High Pure PCR Product Purification Kit (Roche Diagnostics GmbH, Mannheim, Germany) and sequenced using the BigDye^TM^ Terminator v3.1 Cycle Sequencing RR-100, with the same primers (Applied Biosystems, Foster City, CA, USA). The sequencing reaction conditions included preheating at 96°C for 1 min, followed by 25 cycles of 96°C for 10 s, 50°C for 5 s, and 60°C for 1 min. The products were precipitated with ethanol and analyzed by electrophoresis on a 3130xl Genetic Analyzer (Applied Biosystems). Sequence alignment and polymorphism detection were performed using MEGA11: Molecular Evolutionary Genetics Analysis version 11 software [25], and polymorphisms based on the length of the glutamine repeats were confirmed. To verify fragment size, a forward primer labeled with 6-FAM was used. A total of 280 samples (145 males and 135 females) were genotyped using 3130xl Genetic Analyzer (Applied Biosystems), and fragment sizes were estimated based on the GeneScan^TM^ - 400 HD ROX^TM^ Size Standard using the Gene Mapper® Software v4.0 (Applied Biosystems).

### Behavior assessment

Behavioral assessment data were collected from cat owners in Japan using the Feline Behavioral Assessment & Research Questionnaire (Fe-BARQ) [26]. The questionnaire was translated into Japanese by Dr. Nobuyo Ohtani, formerly from Azabu University. It consisted of 101 situation-based behavioral phenotype questions rated on a 5-point scale (0 = never, 4 = always). The responses were standardized into 23 sections with an additional category for “miscellaneous behaviors”.

### Association analysis

Eight alleles reflecting the CAG repeat numbers were observed. These alleles were divided into two groups using the median as a cutoff: alleles with 18 or fewer repeats were categorized as short type (S), and those with 19 or more repeats were categorized as long type (L). In the association analysis, heterozygous females were excluded because one of the X chromosomes was epigenetically inactivated and the chromosomes varied among cells. The association between behavioral traits and genotypes was analyzed using a generalized linear model (GLM), with each of the 23 behavioral sections treated as response variables. Explanatory variables included “genotype” as a factor variable and “age” as a numeric variable; age was categorized according to the 2021 AAHA/AAFP Feline Life Stage Guidelines [27] into: 1, kitten (< 1 yr); 2, young adult (1–6 yrs); 3, mature adult (7–10 yrs); and 4, senior (> 10 yrs). Depending on the distribution of the response variables, a Gaussian error structure with a log link function was applied to “playfulness/activity” and “trainability,” while a gamma error structure with an inverse function was used for the other response variables. To avoid computational issues with the gamma distribution, a constant value of 1 was added to the response variables, which ranged from “never” (1) to “always” (5). Long types (L/- for males and L/L for females) were used as the reference category when constructing the parameter estimates (*ß*) in the GLM. Statistical analyses were performed using the R software version 4.2.3 [28].

### Comparison among Felidae species

To compare polymorphisms among Felidae species, the sequence of the *AR* exon 1 region of a cat (GenBank accession number: AJ893545.1, 1425 bp) was obtained. Homologous regions were identified using a Web BLAST search against the National Center for Biotechnology Information (NCBI) database [29,30]. Only a single sample per species was compared.

## Results

### Genotyping

Eight alleles were identified, ranging from 15 (254 bp) to 22 repeats (275 bp) of the CAG trinucleotide coding for polyglutamines. The total allele frequencies were 0.010, 0.195, 0.154, 0.210, 0.366, 0.051, 0.012, and 0.002 (Table 1).

**Table 1:** Allele frequencies of *AR* in mixed-breed cats.

	Allele frequencies (*n*^a^)							
	Short types				Long types			
	15	16	17	18	19	20	21	22
**Males**	0.021 (3)	0.193 (28)	0.159 (23)	0.221 (32)	0.352 (51)	0.048 (7)	0.007 (1)	0.000 (0)
**Females**	0.004 (1)	0.196 (53)	0.152 (41)	0.204 (55)	0.374 (101)	0.052 (14)	0.015 (4)	0.004 (1)
**Total**	0.010 (4)	0.195 (81)	0.154 (64)	0.210 (87)	0.366 (152)	0.051 (21)	0.012 (5)	0.002 (1)

^a^The number of X chromosomes.

### Association analysis with age and genotype

Age significantly affected several behavioral traits. In males, younger cats exhibited higher scores for “playfulness/activity” (*ß* = -0.413, *p* < 0.001), “touch sensitivity/owner-directed aggression” (*ß* = 0.073, *p* = 0.021), and “crepuscular activity” (*ß* = 0.039, *p* = 0.011), while showing a lower score for “attention seeking” (*ß* = -0.024, *p* = 0.002) (S1 Table). In females, younger cats had higher scores for “playfulness/activity” (*ß* = -0.464, *p* < 0.001) and lower scores for “stranger-directed aggression” (*ß* = -0.123, *p* = 0.031) and “familiar cat aggression” (*ß* = -0.144, *p* = 0.014) (S2 Table).

**Fig 1:**
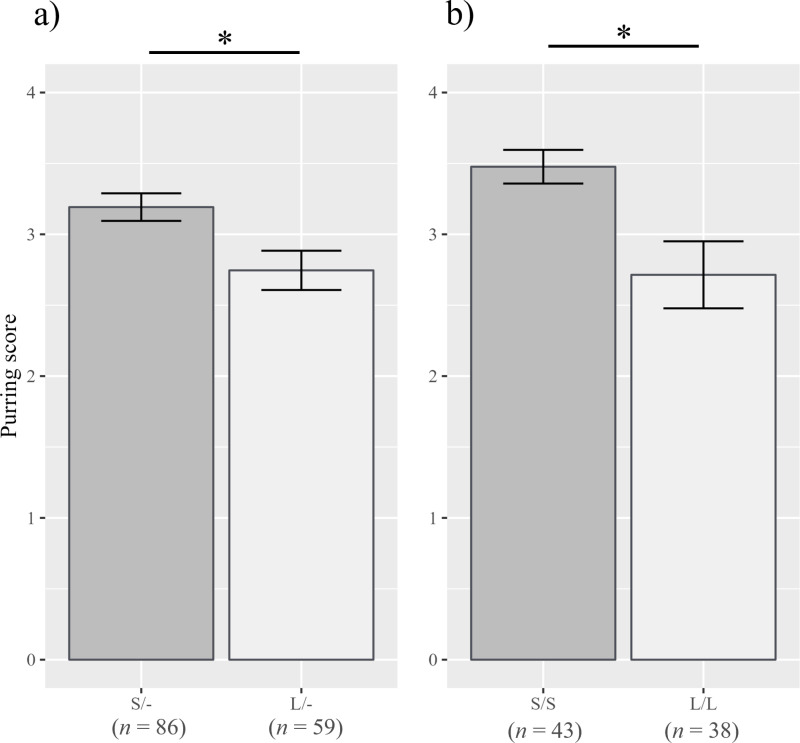
“Purring” scores for each *AR* genotype. (a) Scores for males and (b) scores for females. Error bars represent standard errors, and an asterisk indicates *p* < 0.05.

**Fig 2:**
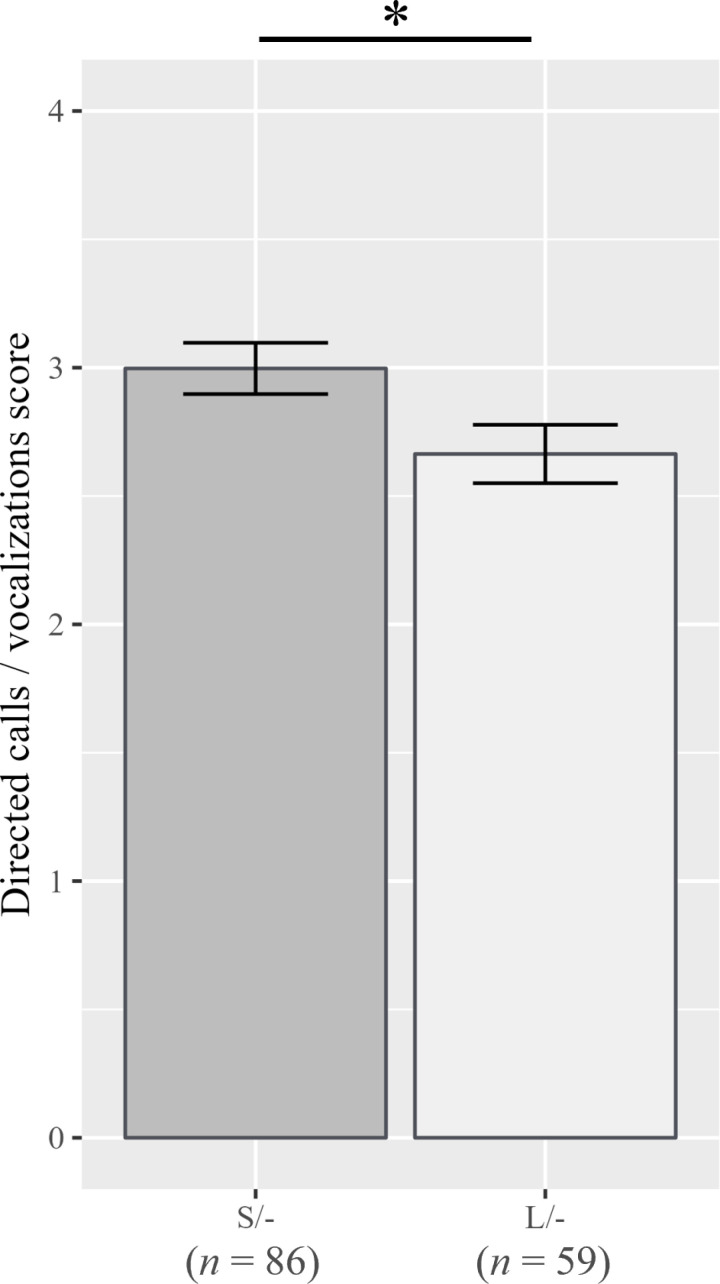
Scores for “Directed calls/vocalizations” for each *AR* genotype in males. Error bars represent standard errors, and an asterisk indicates *p* < 0.05.

**Fig 3:**
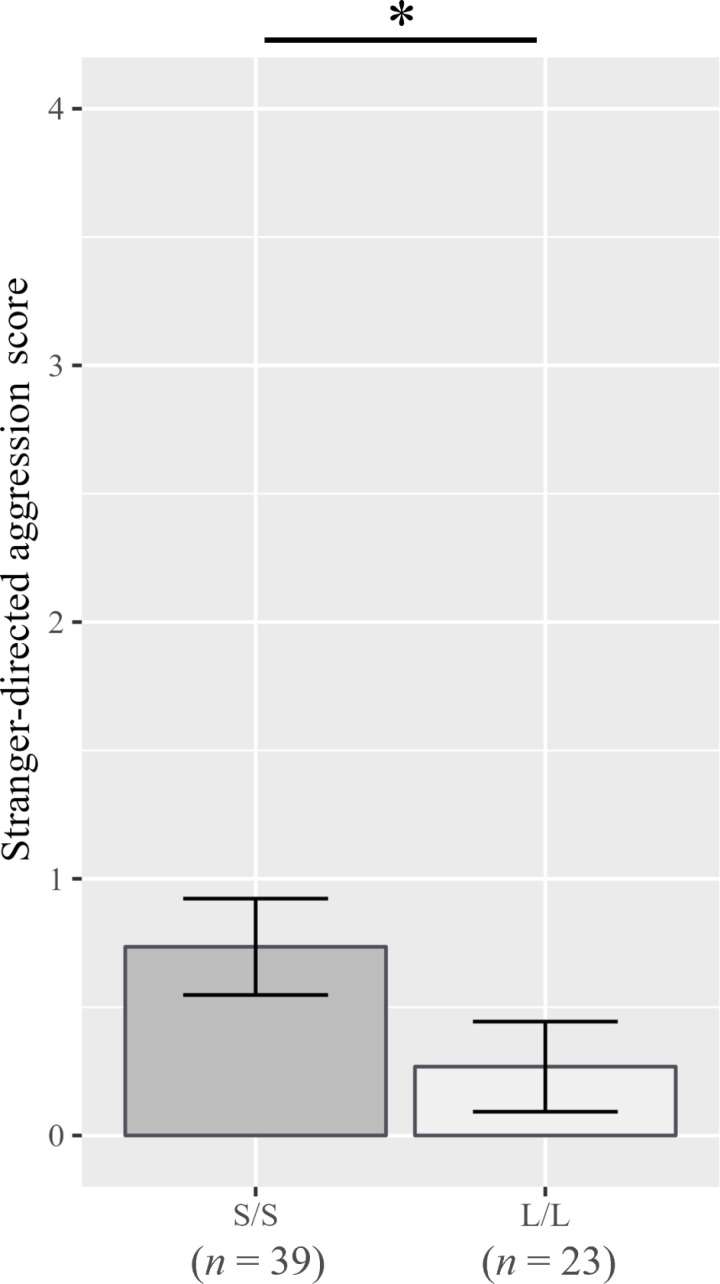
Scores for “Stranger-directed aggression” for each *AR* genotype in females. Error bars represent standard errors, and an asterisk indicates *p* < 0.05.

Significant genotypic effects were observed. Both males and females carrying the short-type (S/- on male, S/S on female) alleles displayed higher “purring” scores (males: *ß* = -0.027, *p* = 0.011; females: *ß* = -0.046, *p* = 0.005; Fig 1) than those carrying long-type alleles. Additionally, males with short-type alleles (S/-) had higher scores for “directed calls/vocalizations” (*ß* = -0.022, *p* = 0.037; Fig 2), whereas females with short-type alleles (S/S) had higher scores for “stranger-directed aggression” (*ß* = -0.244, *p* = 0.040; Fig 3), than the long-type allele-carrying counterparts.

### Comparison with other Felidae species

Homologous regions of the *AR* in cats were found in 11 Felidae species using the Web BLAST search (Table 2). All query coverages were 100%, and percent identity ranged from 95.20% to 98.53%. The glutamine repeat region was conserved across species, with alleles containing 12–19 repeats. Notably, long alleles with 20–22 repeats were observed exclusively in domestic cats. Although the leopard cat lineage is the closest to the domestic cat lineage, this species exhibited only alleles with 15 repeats. Short alleles with 12 and 13 repeats were specific to the Panthera lineage. All species within the Lynx lineage had only 15 repeats, whereas the Puma lineage species exhibited long alleles with 19 repeats observed in the cheetah (*Acinonyx jubatus*).

**Table 2:** Details of Felidae *AR* glutamine repeats.

Species	Scientific names	Repeats	GenBank accession numbers	Lineage
Leopard cat	*Prionailurus bengalensis*	15	XM_043571234.1	Leopard Cat lineage
Fishing cat	*Prionailurus viverrinus*	15	XM_047844059.1	
Jaguarundi	*Puma yagouaroundi*	17	XM_040466962.1	Puma lineage
Cheetah	*Acinonyx jubatus*	19	XM_027053706.2	
Eurasian Lynx	*Lynx lynx*	15	KM368273.1	Lynx lineage
Canada lynx	*Lynx canadensis*	15	XM_030305521.1	
Bobcat	*Lynx rufus*	15	XM_047092579.1	
Geoffroys cat	*Leopardus geoffroyi*	15	XM_045473120.1	Ocelot lineage
Leopard	*Panthera pardus*	13	XM_019428579.2	Panthera lineage
Tiger	*Panthera tigris*	12	XM_042974553.1	
Clouded leopard	*Neofelis nebulosa*	18	XM_058714697.1	

## Discussion

In the present study, glutamine repeat polymorphisms ranging from 15 to 22 repeats were observed in cats. The association analysis of neutered/spayed animals suggested an effect on owner-assessed “purring,” where cats with the short-type alleles displayed higher purring scores than those with longer alleles.

### Behavioral traits and their functions

In our study, purring was associated with *AR* polymorphisms. Male cats with shorter alleles displayed higher “directed calls/vocalizations” scores, which were reflected in the responses to four questions on vocalizations toward humans in the questionnaire. This result aligns with the association between purring and vocal communication as strategies for seeking attention or support, benefiting survival through interactions with both cats and humans.

In female cats, those with short-type alleles displayed higher “stranger-directed aggression” scores. Although aggression may seem distinct from vocalization, this finding is consistent with those of previous studies linking *AR* polymorphisms to aggression or fear [17,19,20]. Given that fear and aggression are often responses to novel stimuli, our findings suggest that aggression in female cats is influenced by similar responses.

### Glutamine repeat numbers and the change of AR function

The function of *AR* glutamine repeat polymorphisms remains unclear. Several studies have suggested that increased glutamine repeats lead to reduced mRNA expression and transactivation [16], whereas one study suggested that medium-length glutamine repeats impart high transactivation [31]. Although the molecular mechanisms are not fully understood, the receptor gene and its main target hormone, testosterone, are linked to behavioral traits. For example, in adolescent boys, a combination of glutamine repeat length and free testosterone has been implicated in depression and its symptoms as well as self-esteem [32,33]. Testosterone also influences vocalization in anurans, birds, rodents, and primates, although the details remain controversial [34]. In wild male chimpanzees, testosterone levels positively correlated with “pant hoot” vocalization rates [35]. As testosterone is a key hormone targeted by AR, changes in *AR* glutamine repeat length could influence testosterone-related behaviors, such as vocal communication. The findings of our study, linking *AR* polymorphisms to vocal behaviors such as purring and directed vocalizations in cats support this observation. Further research on the relationship between testosterone, *AR* polymorphisms, and behavior in cats could enhance our understanding of their physiological and behavioral roles.

### Polymorphism among species and human-animal relationships

We identified 15–22 glutamine polymorphism repeats in cats, consistent with previous reports [21–23].

The comparison among Felidae species in this study confirmed that the longer alleles (20–22 repeats) were unique to domestic cats, while shorter alleles (15 repeats) were observed in the closely related leopard cat lineage. This suggests that longer alleles may be a cat-specific feature, potentially linked to domestication. Furthermore, long alleles are significantly more frequent in pure breeds than in mixed breeds [22], and purebred females exhibit lower heterozygosity than mixed breed females [23], indicating that long alleles have increased in frequency through domestication and selective breeding. Purring surves functions related to affiliation, conflict avoidance, and solicitation [4–6]. Moreover, kitten vocalizations increase maternal behavior [36]. Therefore, purring may be beneficial for feline communication and survival. Given that most mixed-breed cats are rescued former strays—comprising 79% of the sample in this study—we presume that vocalizations may be more critical for survival in mixed-breed cats than in purebred cats bred by breeders. This may have led to a higher frequency of short alleles in mixed-breed cats, whereas in purebred cats, the availability of human care could reduce the need for vocalizations, allowing long alleles to persist.

Similar trends, which supports the hypothesis that domestication enhances the frequency of specific alleles, have been observed in other species. For example, in dogs’ *AR* gene, longer alleles linked to lower aggression are more frequent in European dog breeds than in Chinese or European wolves (*Canis lupus*) or in Asian dog breeds [19,37]. Thus, the way of domestication and the specific roles of dogs in different societies (e.g., guarding in Asia vs. herding in Europe) likely influence the prevalence of aggression-related genes [38]. In camels, short-type alleles of the *AR* gene are associated with fear [20]. Domesticated camel breeds raised for agricultural purposes (e.g., Baladi and Maghrabi) have fewer short alleles than those raised in large groups in natural pastures (e.g., Sudani and Somali) [20]. Interestingly, Asian elephants (*Elephas maximus*), which are partially domesticated, possess a shorter allele than African elephants (African savannah (*Loxodonta africana*) and African forest (*L. cyclotis*) elephants) do, further demonstrating the role of human selection in allele frequency variation, although its relationship to behavioral traits has not been observed [38,39]. Furthermore, shorter *AR* alleles are more common in domesticated horses (*Equus caballus*) than in the zebra species Grevy’s zebras (*Equus grevyi*), plains zebras (*Equus quagga*), and Hartmann’s mountain zebras (*Equus zebra hartmannae*) [40]. Thus, these results align with the findings in camels, where shorter alleles are linked to fear. Considering that they are ungulates, horses with shorter alleles might be more fearful and more inclined to seek human protection, resulting in a recent human-horse relationship [38]. Similarly, cat domestication and the generation of modern cat breeds may have influenced the evolution of behavior-related genes, as the long-type *AR* allele—suggested in this study to be related to lower “stranger-directed aggression”—is more frequently observed in pure breeds than in mixed breeds [22]. However, these observations are based on single-gene comparisons. More comprehensive whole-genome studies are necessary to fully understand the effects of domestication on genetic evolution. Further research is crucial to clarify the broader implications of human-animal relationships on genetic and behavioral traits.

## Limitations

The samples used in this study were limited to mixed-breed cats housed in Japan. Mixed breeds have a high genetic diversity [41], making them suitable for the detection of various genotypes. However, pure breeds tend to form several genetic clusters [41–44], and breed differences have been observed in behavioral traits, such as aggression and vocalization [45,46]. Thus, breed-specific characteristics may have introduced some bias into this study. Given that mixed breeds are the most popular cat breed in Japan as of 2023 [47], studying them can provide insights into the characteristics that are applicable to a large portion of the cat population. Additional research on genetic differences among pure breeds is needed to deepen our understanding of breed-specific behavioral traits and their development.

We evaluated behavioral traits using owner-reported questionnaires, which may have included the subjective views of the owners. Although such questionnaires are useful for collecting large amount of data required for genetic analyses, particularly when working with diverse genotypes, owners tend to evaluate their pets more favorably than others [48]. One advantage of using questionnaires is that they allow for the assessment of everyday behaviors in a familiar home environment without the introduction of new stimuli, such as the presence of researchers or unfamiliar experimental settings. Supplementing questionnaire data with behavioral tests would provide a more robust approach for confirming specific behavioral traits.

The comparison of Felidae species in the present study relied on database records; thus, the analysis was constrained by limited sample size and species representation, with some species, such as lions that live in social groups akin to cats, not being included because of the lack of available information. Additionally, genetic polymorphisms may vary across Felidae species, potentially leading to genotypic differences. This study highlighted the influence of glutamine repeats in *AR* functioning, suggesting that expanding the sample size and species representation in future studies could provide deeper insights into feline behavioral traits and domestication processes.

## Conclusions

The association analysis of genotype and behavioral traits revealed that short alleles of *AR* were associated with increased purring in cats. Variations in allele lengths were also observed across various Felidae species. The findings from our study have potential applications in improving animal welfare by predicting behavioral tendencies based on genetic data and facilitating need-based observation and enhanced care. For example, cats with longer alleles may be less vocal, and the risk of overlooking their health conditions or distress in the absence of vocal distress signals could be mitigated. These findings are beneficial not only for cats but could also be extended to other Felidae species. For example, estimating the behavior of rescued animals using genetic information could help create more suitable housing environments and offer better care, especially in the context of ex situ conservation programs. Future research on behavioral traits would contribute to enhancing human—cat relationships and conservation.

## Supporting information

S1 Dataset Profiles of genotyped samples. (CSV)

S1 Table Generalized linear model results from male cats. (XLSX)

S2 Table Generalized linear model results from female cats. (XLSX)
